# Calibration and performance of a Raman-based device for non-invasive glucose monitoring in type 2 diabetes

**DOI:** 10.1038/s41598-025-95334-x

**Published:** 2025-03-25

**Authors:** Anders Pors, Barbara Korzeniowska, Markus T. Rasmussen, Christian V. Lorenzen, Kaspar G. Rasmussen, Rune Inglev, Amalie Philipps, Eva Zschornack, Guido Freckmann, Anders Weber, Karl D. Hepp

**Affiliations:** 1 RSP Systems, 5260 Odense, Denmark; 2 Institute for Diabetes Technology, University of Ulm, 89081 Ulm, Germany; 3 University of Munich (Emeritus) and Forschergruppe Diabetes, 85764 Oberschleissheim, Germany

**Keywords:** Non-invasive glucose monitoring, Raman-based sensor, Sensor calibration model, In-vivo measurements, Interstitial compartment, Diabetes management, Endocrine system and metabolic diseases, Optical sensors, Raman spectroscopy

## Abstract

Raman spectroscopy has been demonstrated as a viable technique for non-invasive glucose monitoring (NIGM). However, its clinical utility is limited by an extended calibration period lasting several weeks. In this study, we address this limitation by employing a pre-trained calibration model, which is individualized through a brief calibration phase consisting of 10 measurements. The performance of the Raman-based NIGM device was evaluated in a clinical trial involving 50 individuals with type 2 diabetes over a 2-day study period. The protocol included a 4-h calibration phase on the first day, followed by validation phases of 4 h and 8 h on days 1 and 2, respectively. NIGM glucose readings were compared with capillary blood glucose measurements, with glucose fluctuations induced by standardized meal challenges. The numerical and clinical accuracy of the NIGM device was evaluated on 1918 paired points and expressed by mean absolute relative difference of 12.8% (95% CI 12.4, 13.2) and consensus error grid analysis showing 100% of NIGM readings in zones A and B. These results highlight the ability to reliably track blood glucose levels in people with type 2 diabetes. The successful introduction of a practical calibration scheme underlines Raman spectroscopy as a promising technology for NIGM and constitutes an important step towards factory calibration.

**Supplementary Information:**

The online version contains supplementary material available at 10.1038/s41598-025-95334-x.

## Introduction

Since the advent of glucose self-monitoring in the 1970s, non-invasive glucose measurement has been a hotly desired methodology with the aim to overcome pain and discomfort of multiple finger prick tests. During the past decades a variety of different technologies were developed and tested but to date, none of these has gained full clinical and practical acceptance^1,2^. In the meantime, the minimally invasive electrochemical technology of continuous glucose monitoring was successfully introduced into glucose monitoring in diabetes, heralding a new philosophy of glucose control in the interstitial compartment^3,4^.

Among the many approaches to truly non-invasive methods, optical techniques, coupled with multivariate statistics, gain most of the attention due to the wealth of information residing in spectroscopic data^5^. Current efforts encompass radio and microwave frequency sweeps^6–8^, photo-thermal or -acoustic spectra using mid-infrared light sources^9,10^, and absorption spectroscopy with near-infrared light^11^. Some of these technologies are attractive with respect to cost, size, and simplicity but the development is still in the early stages, so it is uncertain whether glucose specificity and signal-to-noise ratio are sufficient for stable and accurate glucose monitoring under real-world conditions. The situation is different with near-infrared Raman spectroscopy, which since the first feasibility study in 1997^12^ has been thoroughly validated and now considered one of the most promising technologies for non-invasive glucose monitoring (NIGM)^13,14^. In contrast to other spectroscopic methods, Raman is insensitive to water in the molecular fingerprint region, which makes it particularly suitable for in-vivo applications^15^, and, more importantly, it directly probes the glucose molecules in the interstitial compartment^16,17^. The main challenge is the intrinsically weak Raman signal, which necessitates a relatively complex instrumentation and intense laser source, thus precluding a simple path towards miniaturization. The current development in light sources, micro- and nano-optics, and detectors^18–20^, however, open for shrinking Raman systems to wearable form factors^21^—a prerequisite for entering the smartwatch and diabetes management markets.

RSP Systems has used the last decade to design, refine, and test a portable Raman spectroscopy system that is optimized for NIGM in the interstitial compartment of the skin. The NIGM device is a result of systematic clinical testing, including real-life assessment in out-patient settings^22^ and performance evaluation in blinded in-clinic studies^23^. In the last home-based clinical study, encompassing 160 individuals with type 1 and type 2 diabetes, it was demonstrated that the Raman-based system meets the necessary criteria for performance and effectiveness as a device, with a measurement accuracy that is insensitive to age, sex, and skin color, and a calibration that can be sustained for a minimum of 15 days^24^. The study was based on an extended calibration period of 26 days (6 measurements per day), which may be considered impractical in a real-life scenario, but a requirement imposed by multivariate calibration models when trained without a priori knowledge of the intricate relationship between skin Raman spectra and blood glucose concentrations^25^.

The present study was designed for two main purposes. Firstly, to test a new calibration scheme that utilizes a pre-trained calibration model, thereby reducing the calibration requirement to 4 h (10 measurements), and, secondly, to evaluate the device’s ability to follow meal-induced glucose excursions in individuals with type 2 diabetes. The study encompasses 50 individuals with an intended variability in age, BMI, duration of diabetes, and form of therapy.

## Materials and methods

### Clinical study overview

The study was performed as an explorative, non-randomized, in-clinic trial of the NIGM device at Institut für Diabetes-Technologie Forschungs- und Entwicklungsgesellschaft mbH an der Universität Ulm, Germany. All procedures conducted in this study adhered to the ethical standards set by the institutional and national research committees, as well as the principles outlined in the 1964 Helsinki Declaration and its subsequent amendments. The study was approved by the German Federal Institute for Drugs and Medical Devices with the unique EUDAMED identifier: CIV-23-02-042313 and registered on ClinicalTrials.gov (NCT05851469) on 09/05/2023. Informed consent was obtained from all participants prior to their inclusion in the study.

### Device under study

**Fig. 1:**
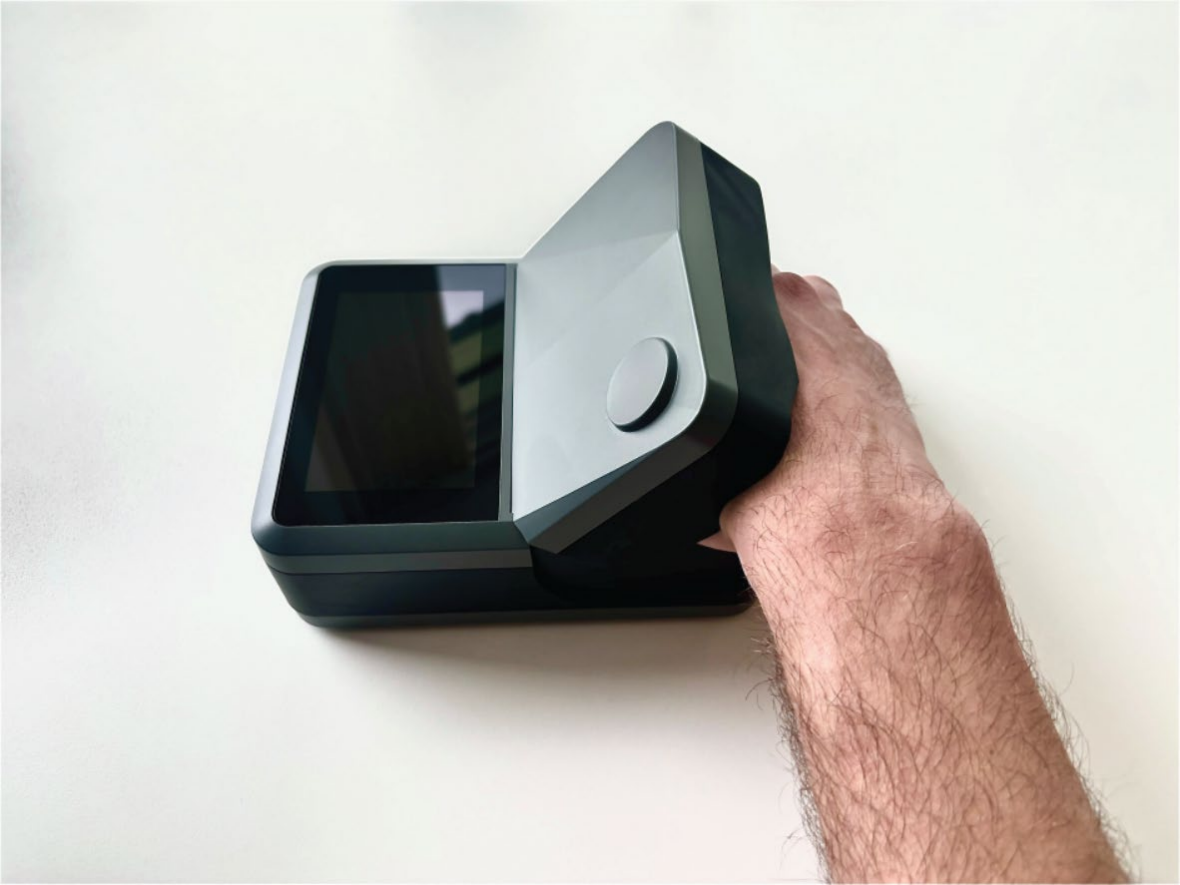
A digital image of a novel, portable, stand-alone, Raman-based device configured for NIGM.

The NIGM device, illustrated in Fig. 1, is a stand-alone, portable (168 mm (l) × 130 mm (w) × 62 mm (h)), Raman-based system equipped with built-in safety measures, graphical user interface, and Wi-Fi connectivity. The base of the thumb (i.e., thenar) is illuminated with 300mW laser light at a wavelength of 830 nm, and the backscattered light is collected, filtered, and dispersed using a spectrometer with a spectral resolution of ~ 10 cm^−1^ in the spectral range 300 to 1615 cm^−1^. The device is configured to mainly collect the signal from the upper, living part of the skin, while the signal from the outer, dead skin layer (stratum corneum) is suppressed. For further technical details of the device, we refer to Ref.^24^, which contains a thorough description of the optical configuration and associated specifications.

### Patients

50 subjects with type 2 diabetes were recruited for this study. They were familiar with blood glucose self-monitoring (BGSM) and instructed in the use of the NIGM device. A broad variability in clinical characteristics was intended. For eligibility criteria, see Supplementary Materials. Skin phototype was assessed according to the Fitzpatrick scale^26^ and by utilizing a skin tone sensor (DEESS Demi II, Shenzhen GSD Tech Co., Ltd) on dorsum of hand, and skin thickness on right hand thenar was determined by optical coherence tomography (Callisto, Thorlabs)^27^.

### Study design

The study was distributed over 2 consecutive days with 24 and 26 measurements performed on day 1 and day 2, respectively (for an overview, see Supplementary Fig. 1). The first 10 measurements on day 1 constituted the calibration sessions composed of 1 venous blood sample (Cobas Integra 400, Roche), 2 capillary blood samples for BGSM (Contour Next, Ascensia Diabetes Care) and 4 hand placements on the NIGM device, each entailing 50 s of spectral data collection. The following 14 and 26 validation sessions performed on study day 1 and study day 2, respectively, included 2 capillary blood samples and 4 hand placements on the NIGM device per session. During the validation measurements performed on study day 1, a venous blood sample was drawn every 30 min, corresponding to every second validation session. Calibration and validation measurements occurred at intervals of 20 and 15 min, respectively, with breaks and meals distributed throughout the days. Participants were served breakfast and lunch with high carbohydrate content to induce post-prandial glucose excursions.

The glucose reference values used to evaluate the device accuracy were derived from the capillary blood samples and were an average of two consecutive measurements, while the corresponding NIGM measurements were an average of the four hand placements. The subset of measurements on study day 1 with both capillary and venous values enabled studying the influence of the reference method on the accuracy of the NIGM device.

The study included ten NIGM devices, with five subjects on each. The use of multiple devices allowed for conducting the study efficiently and with five subjects in parallel (maximum capacity at the study site). A statistical evaluation of the device’s performance was not intended.

### Data analysis

**Fig. 2:**
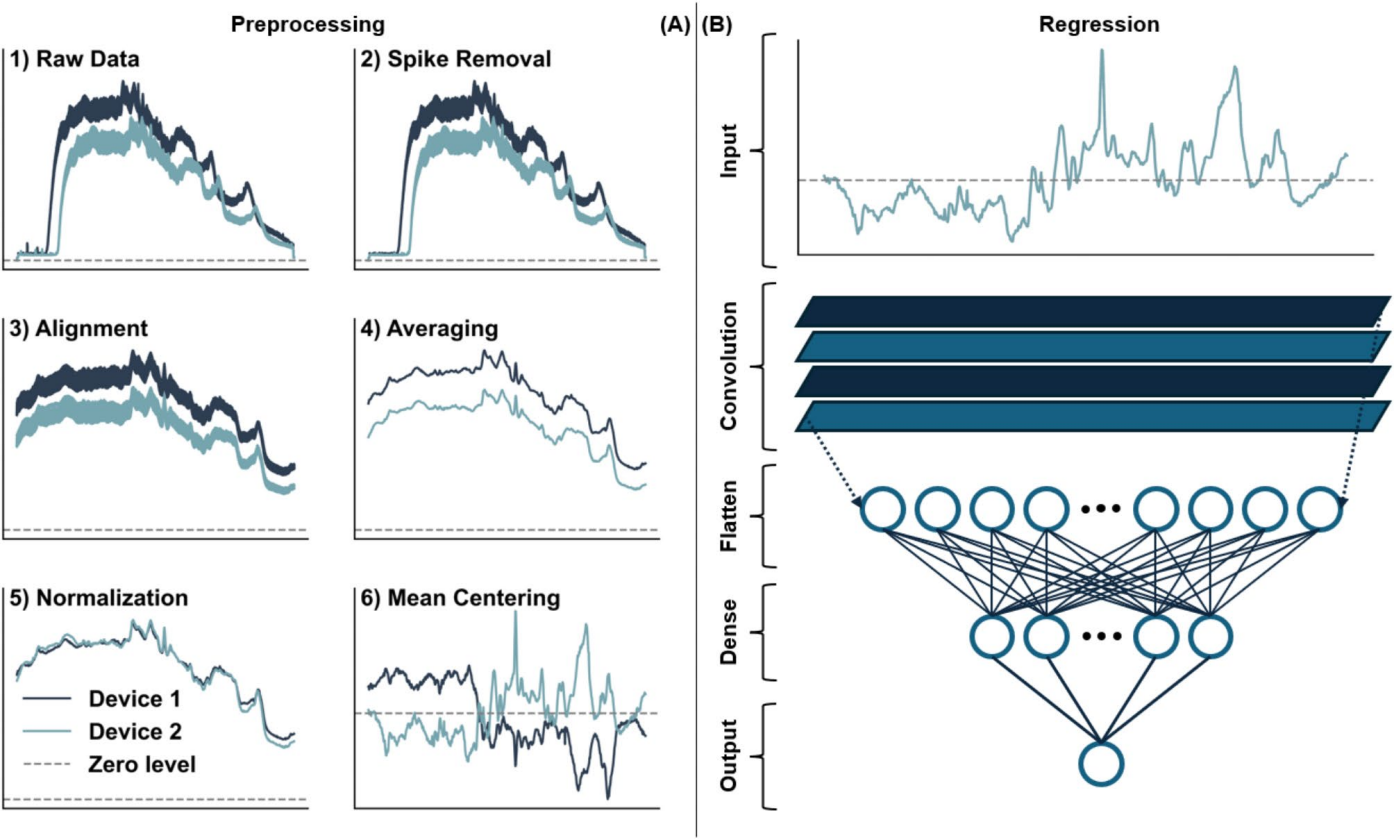
(**A**) Illustration of the preprocessing steps involved in preparing the spectral data for the regression model. The panels show spectra from two devices. (**B**) The regression model is a pretrained convolutional neural network that calculates the glucose concentration from the (preprocessed) spectral input.

The calculation of blood glucose concentrations from recorded skin Raman spectra involved two core elements: preprocessing of spectra and the pretrained regression model. The two elements are depicted in Fig. 2. Preprocessing is essentially a way of standardizing the recorded spectra to assist the regression model in quantifying the glucose content in the spectra. Figure 2A illustrates the preprocessing steps for two hand placements (50 s measurement series) from two different devices. Each hand placement on the device brought about a series of raw spectra that were represented by 1024 pixel values (step 1). These spectra were then cleaned by removing spikes (step 2) that arose from flickering/hot pixels and interference from cosmic rays. The spectra were subsequently aligned to the common Raman axis (300-1615 cm^−1^) in 700 equidistant points (step 3), followed by spectral averaging (step 4) to improve signal-to-noise ratio. The remaining two steps involved normalization (step 5) and mean-centering (step 6) to obtain similar-looking spectra that vary around the zero line.

The pretrained calibration model, as depicted in Fig. 2B, was a shallow one-dimensional convolutional neural network (CNN) consisting of a single convolutional layer, a flatten layer, a single dense layer, and the final one-neuron output layer containing the derived glucose value. The CNN model featured 105 k trainable parameters, which is a small number compared to other studies that combine spectral data and CNNs^28,29^. The small network, combined with a large training set of ~ 110 k paired spectra and reference glucose values, ensured a pretrained calibration model that generalized well to new, unseen data. The calibration data of the study participants, collected in the morning of study day 1, were used to fine-tune the pretrained model to the individual subjects. Note that training data were from an earlier large-scale, clinical study involving 160 subjects (for details, we refer to Ref.^24^) and, as such, were completely decoupled from the current study. Data were analyzed by RSP Systems using Python 3.10.9 (Numpy 1.23.5 and Tensorflow 2.10.0).

The study was planned to nominally include 2000 validation points (40 validation points per subjects). However, two factors reduced the final number of validation points to 1918 (see Fig. 4). The first reason was malfunctioning of two devices, resulting in the loss of 50 validation points. The second factor owes to removal of reference outliers, which were defined by a difference larger than 1 mmol/L between the two consecutive BGSM measurements. No spectral outlier check was implemented during data analysis as on-device algorithms ensured the quality of recorded Raman spectra.

### Objectives

The primary outcome was to assess the clinical performance of a pre-trained calibration model by Consensus Error Grid (CEG) analysis as well as the accuracy metrics Mean Absolute Relative Difference (MARD) and Root Mean Squared Error (RMSE). The secondary outcome was to explore possible effects of user-dependent parameters (see Table 1) on the performance of the NIGM device. Correlations between MARD and user-dependent parameters were determined by Pearson correlation.

**Table 1:** Clinical characteristics of participants.

Variable		Data value (*N* = 50)
Age (years)		64 (38–83)
Male/Female		28/22
BMI (kg/m^2^)		28.0 (22.3–47.5)
Duration of diabetes (years)		15 (2–40)
HbA1c (mmol/mol)		55 (36–95)
Treatment	Basic (i.e.: diet, exercise, training)	2
	Oral antidiabetics	44
	Subcutaneous antidiabetics	13
	Basal insulin	11
	Conventional insulin therapy	9
Skin phototype I/II/III/VI/V/VI		0/3/22/24/1/0
Ethnicity: Caucasian/Others		50/0
Stratum Corneum thickness (µm)		163 (89–315)

Continuous data are presented as median (range), while categorial data are presented in absolute numbers of participants. Skin phototype is based on the Fitzpatrick scale, while the stratum corneum thickness on thenar is measured using optical coherence tomography. Note that participants can be on multiple treatments.

## Results

Table 1 shows the clinical characteristics of the 50 participants under study with the intentional diversity in age, BMI, duration of diabetes, HbA1c, and form of therapy, to cover a wide spectrum for testing the performance of the device. As relevant for any optical technique that probes the glucose content in the interstitial compartment of the skin, the table also includes information on the participant’s general skin color, ethnicity, and stratum corneum thickness. The median thickness of the stratum corneum was 163 µm (with a range of 89–315 µm) which must be considered in relation to the NIGM device’s nominal collection depth of 280 µm.

**Fig. 3:**
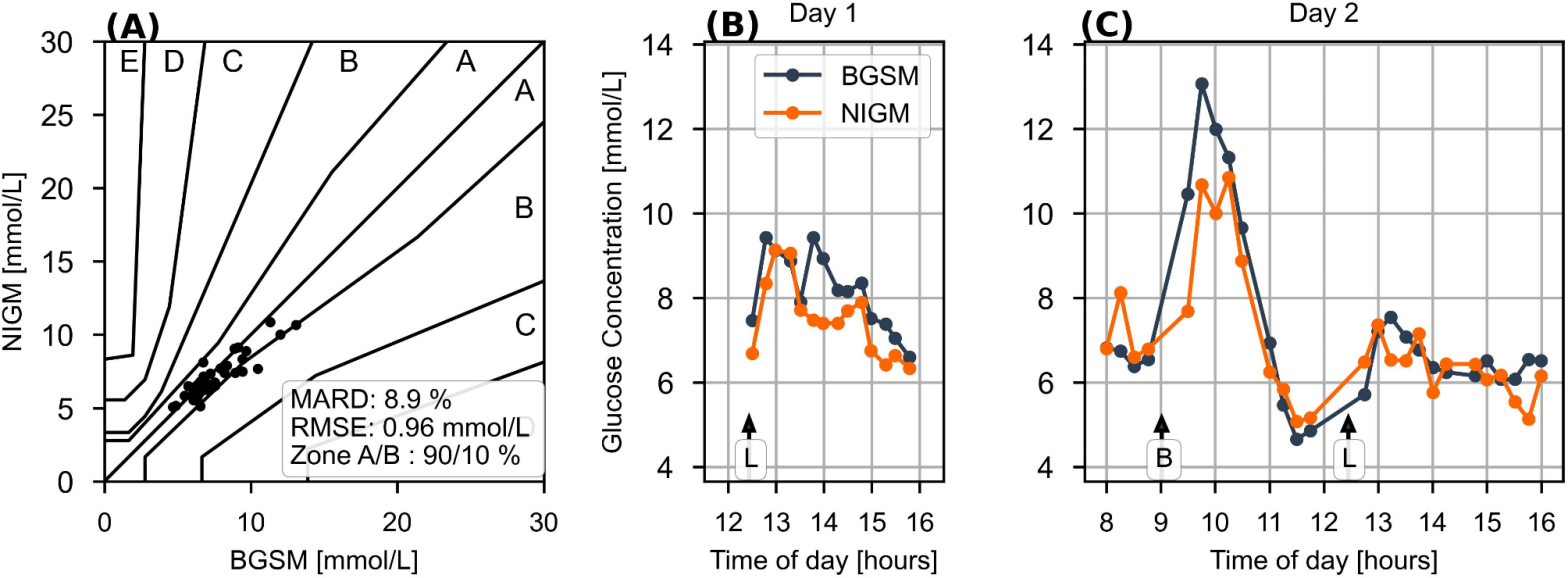
Example of a participant’s BGSM and NIGM profiles during 2 days of in-clinic validation under test meal challenges. The participant is a 60-year-old woman with a HbA1c of 48.0 mmol/mol, and she is one of the best performing individuals. (**A**) Consensus error grid, demonstrating 90.0% of points in zone A. (**B**,**C**) Time-course of BGSM and NIGM measurements during the validation phase. Breakfast and lunch are indicated with B and L, respectively.

To approach continuous glucose kinetics, we chose 15 min intervals for consecutive glucose determinations which ensures faithful tracking of the meal-induced glucose profiles, practical realizable with the sequence of BGSM and NIGM measurements (including venous blood samples every 30th min on day 1), and tolerable for subjects over an extended measurement period of 8 h per day. Figure 3 exemplifies the performance and time course of NIGM versus BGSM measurements during meal challenges on the two study days in a subject. Glucose excursions show the delay for the interstitial concentration in the upswing, in this subject estimated to 6 min. Closeness between capillary and non-invasive glucose concentrations is expressed by RMSE of 0.96 mmol/L, MARD of 8.9%, and 90.0% of points in zone A of the CEG plot.

**Fig. 4:**
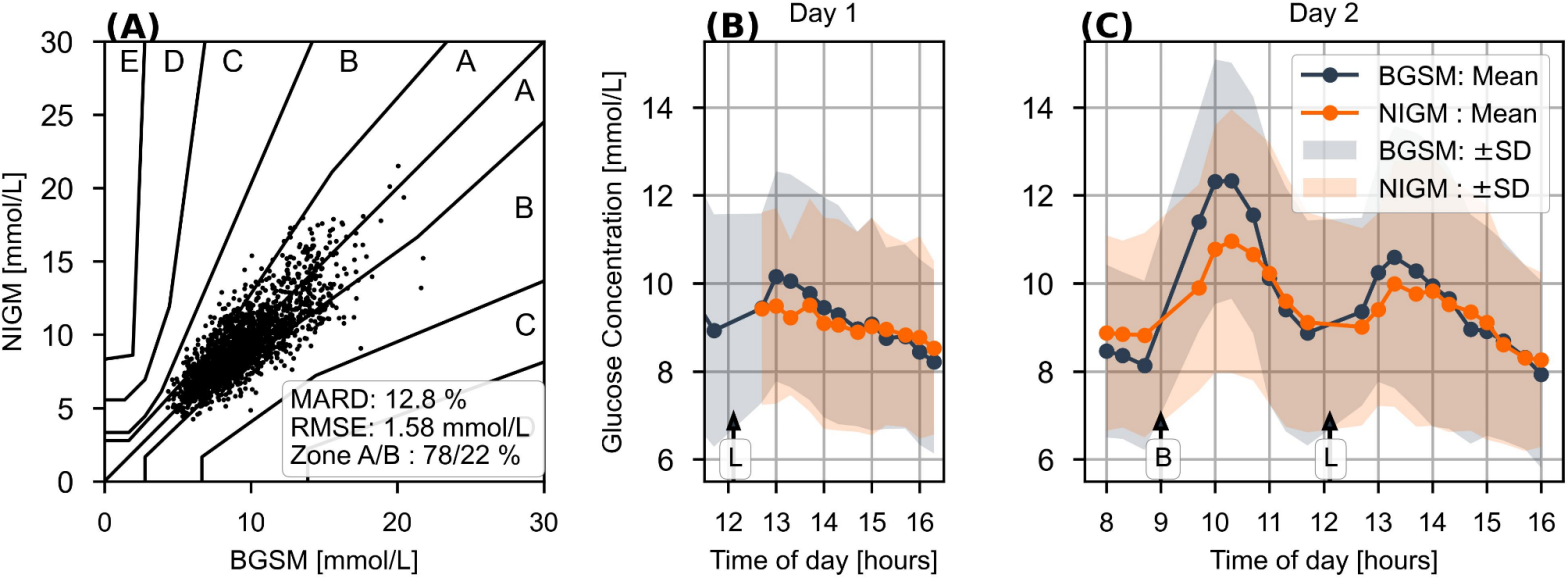
Aggregated performance of 50 individuals. (**A**) CEG plot of the 1918 validation points. (**B**,**C**) Time course of average BGSM versus NIGM values during the validation period. Shaded areas correspond to ± SD. Breakfast and lunch are indicated with B and L, respectively, and correspond to an average carbohydrate intake of 70 g and 80 g.

Figure 4 shows the aggregated performance and mean glucose excursions for the entire cohort of 50 subjects during the validation phase (corresponding calibration data presented in Supplementary Fig. 2). The delay in the upswing appears longer than that of the better-performing subject in Fig. 3, indicating variability in glucose diffusion between capillary and interstitial space. By delaying the individual BGSM curves for optimizing the correlation with the NIGM measurements, the average interstitial glucose delay is estimated to 10 min with a variability expressed by a standard deviation of 9 min. These numbers should be compared to CGM systems with an estimated time delay of 9.5 ± 3.7 min^30^, hence highlighting a larger variability for the NIGM device. The overall NIGM performance is characterized by the indices for RMSE of 1.58 mmol/L (95% CI 1.53, 1.64) and MARD of 12.8% (95% CI 12.4, 13.2) for all 50 patients. The CEG plot shows 100% of points within zones A and B with 63.4% and 36.6% of paired measurements distributed in the eu- and hyper-glycemic ranges, respectively. No data were obtained from the hypoglycemic range (< 3.9 mmol/L).

**Fig. 5:**
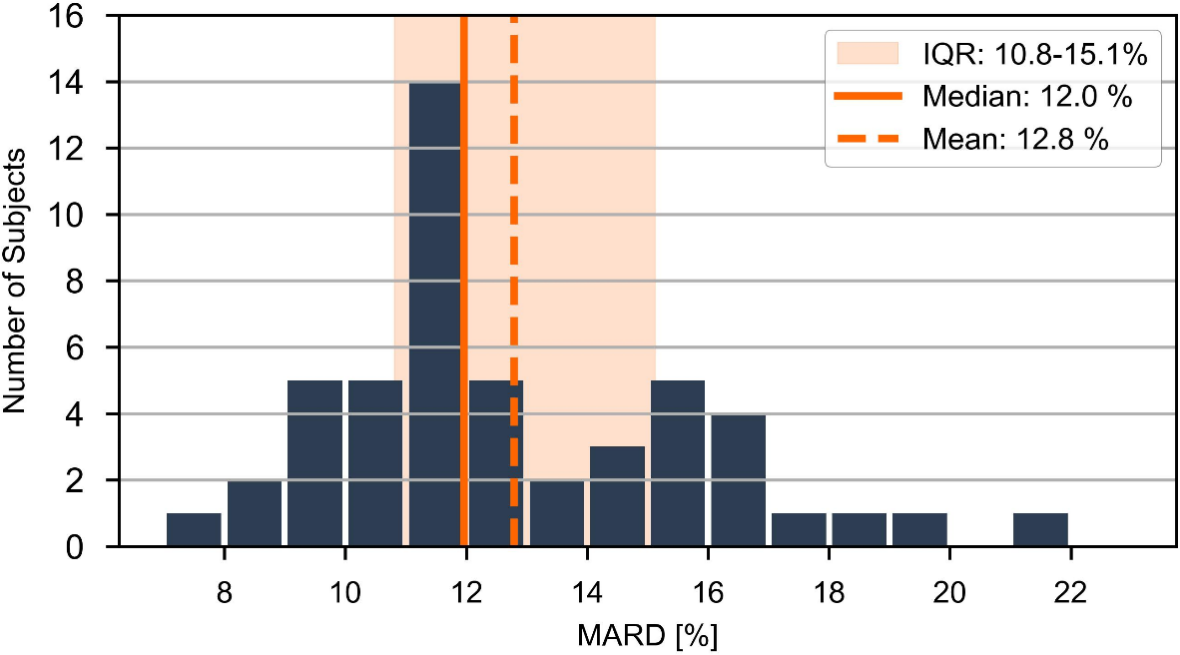
Histogram of the 50 participant’s MARD values, assessed over the ~ 1.5 days validation period. Several statistical parameters (mean, median, and interquartile range (IQR)) are highlighted in the figure.

Figure 5 indicates the variability in individual performance, highlighting a noticeable span in recorded MARD values (7.1–21.8%). The median MARD value, representing the threshold between the 50% best and worst performing individuals, is 12.0% (95% CI 11.4, 12.8). We have investigated whether the observed MARD values correlate with patient characteristics in Table 1, however no significant correlations were found (Supplementary Table 1). With the knowledge that skin Raman spectra feature an increased level of fluorescence background for darker skin types^24^, it is comforting to see that skin phototype (r = 0.26, *p* = 0.07) does not strongly influence performance, which is in line with earlier findings^24^ and implies that measurement error is not dominated by shot-noise.

## Discussion

The current study indicates the consistent development towards a convenient, Raman based device for non-invasive glucose monitoring.

Whereas our previous studies have shown the gradual improvement in hardware and real-life performance^22–24^, the long calibration phase has limited practical utility^24^. By introducing a pre-trained calibration model based on past clinical data, we were now able to reduce the number of calibration points to 10, carried out in the morning. More important, our retrospective analysis indicates that performance begins to converge after 6 calibration measurements (Supplementary Fig. 3), hereby highlighting the ability to further reduce the calibration requirement for the convenience of the user.

It is worth noting that despite the in-clinic setting and the frequent measurements, the reported NIGM values are based on intermittent (i.e., uncorrelated) measurements without mathematical correction for glucose kinetics and noise reduction by time-series filtering, which were found to improve performance metrics in electrochemical CGM sensors^31^. The possibility to further improve performance through time-correlated measurements is a topic relevant for a future wearable device that allows for uninterrupted, semi-continuous measurements.

Overall accuracy of the NIGM device, as represented by MARD of 12.8% and 100% of points in zones A and B of the consensus error grid in the cohort of 50 patients with type 2 diabetes, is encouraging for a device in development. The current prototype has the potential for use in type 2 diabetes and stages of increased metabolic risk. Yet, accuracy, as defined by MARD and RMSE, is not solely dependent on technical quality of the NIGM device, but as in the case of CGM, also on factors such as accuracy of the reference device, compartment differences, patient characteristics, and study design^32^. In the current study, we investigated the influence of the reference method on the device performance. The analysis is based on a subset of measurements on day 1 (see Supplementary Fig. 1) and it revealed a ~ 11% and ~ 7% improvement in RMSE and MARD, respectively, when the reference glucose values derive from the laboratory Cobas system (based on venous blood samples) in comparison with the home-use Contour Next device (based on capillary blood samples). The observed improvement in measurement accuracy, which is detailed in Supplementary Fig. 4, can be ascribed to two factors. Firstly, the post-prandial glucose dynamics in venous blood, being slightly delayed and at lower values compared to the capillary counterpart^33^, better reflects the glucose content in the interstitial compartment. Secondly, the laboratory-grade Cobas system is noticeably more accurate than the home-use Contour Next device, hence entailing that the accuracy of the NIGM device is less penalized by reference uncertainty when using the Cobas system. For further details on the systematic difference between blood glucose values in the capillary and venous compartments and measurement uncertainty of the two reference systems, we refer to Supplementary Fig. 5.

The current study is characterized by a 2-day in-clinic setting, the use of a BGSM comparator, patients with type 2 diabetes, and glucose excursions induced by standard meals. Limitations are that the study design prevented glucose values to reach the hypo-glycemic range, hence precluding the possibility to gauge the measurement accuracy in this clinically important range. Also, it awaits to be seen whether the current performance can be sustained in individuals with type 1 diabetes and in subjects of different ethnic backgrounds, as all study participants were Caucasian. Earlier results have indicated that the larger and faster glucose excursions typically seen in people with type 1 diabetes negatively impact performance metrics^24^. Finally, the limited study duration could not deal with long term calibration stability. It is, however, comforting to note that past results have shown sustained calibration for a minimum of 15 days^24^, thus indicating that current results are expected to hold for weeks before the need of a re-calibration point, if any at all.

In conclusion, the successful application of a practical calibration scheme underlines Raman spectroscopy as a promising technology for NIGM in diabetes management. Through collection of more data in upcoming studies, it is expected that the pre-trained calibration model can be refined, leading to improved measurement accuracy, less variability between subjects, and a further reduction in calibration requirement.

## Electronic supplementary material

Below is the link to the electronic supplementary material.

Supplementary Material 1

## Data Availability

Anonymized data from the current study are available from the corresponding author upon reasonable written request.

## Declarations

### Prior presentation

Parts of this study were presented as a poster presentation at the 17^th^ International Conference on Advanced Technologies & Treatments for Diabetes (ATTD 2024), Florence, Italy, 6–9 March 2024.
