# Supplementary material for: Calibration and performance of a Raman-based device for non-invasive glucose monitoring in type 2 diabetes
Source: Sci Rep. 2025 Mar 25;15:10226. doi: 10.1038/s41598-025-95334-x (PMC11937273; doi:10.1038/s41598-025-95334-x)
Supplement: Supplementary file 1 — Supplementary Material 1 [file 41598_2025_95334_MOESM1_ESM.pdf]

Calibration and Performance of a Raman-based Sensor for Non-invasive Glucose Monitoring in Type 2 Diabetes

Supplementary materials

Eligibility criteria

Supplementary Figure 1: Schematic presentation of the 2-day clinical study, highlighting the calibration and validation phases, meals, and breaks.

Supplementary Figure 2: Time-course of glucose values during the 4-hour calibration phase in the morning of day 1.

Supplementary Figure 3: Pooled measurement performance vs number of calibration measurements.

Supplementary Figure 4: Pooled glucose measurements for two reference methods.

Supplementary Figure 5: Reference glucose values in venous and capillary blood.

Supplementary Table 1: Correlation between participant characteristics and MARD values.

Eligibility criteria

Inclusion criteria: male or female subject ≥18 years, subject with type 2 diabetes, signed consent form informed as expected by the Helsinki Declaration with subsequent amendments.

Exclusion criteria: intensified insulin treatment; elevated level of sodium, potassium, creatinine, eGFR, and ALAT (more than three times the upper limit of normal range); clinically relevant anaemia or thrombocytopenia; infection with hepatitis B, C or HIV; intake of anticoagulant medication (with the exception of acetylsalicylic acid); pregnancy or lactation period; subjects not able to understand and read local language; inability to hold arm or hand still (including tremors and Parkinson’s disease); skin changes, tattoos or diseases on right thenar (measurement site); reduced circulation in right hand evaluated by Allen’s test; known allergy to medical grade alcohol (used to clean the skin); haemodialysis; systemic or topical administration of glucocorticoids at the right hand for the past 7 days; inability to comply with the study procedures (due to, e.g., psychiatric diagnoses, lack of cognitive ability, alcohol dependency, drug use, or psychosocial overload); dependence from the sponsor or the clinical investigator; participation in another study.

Supplementary Figure 1

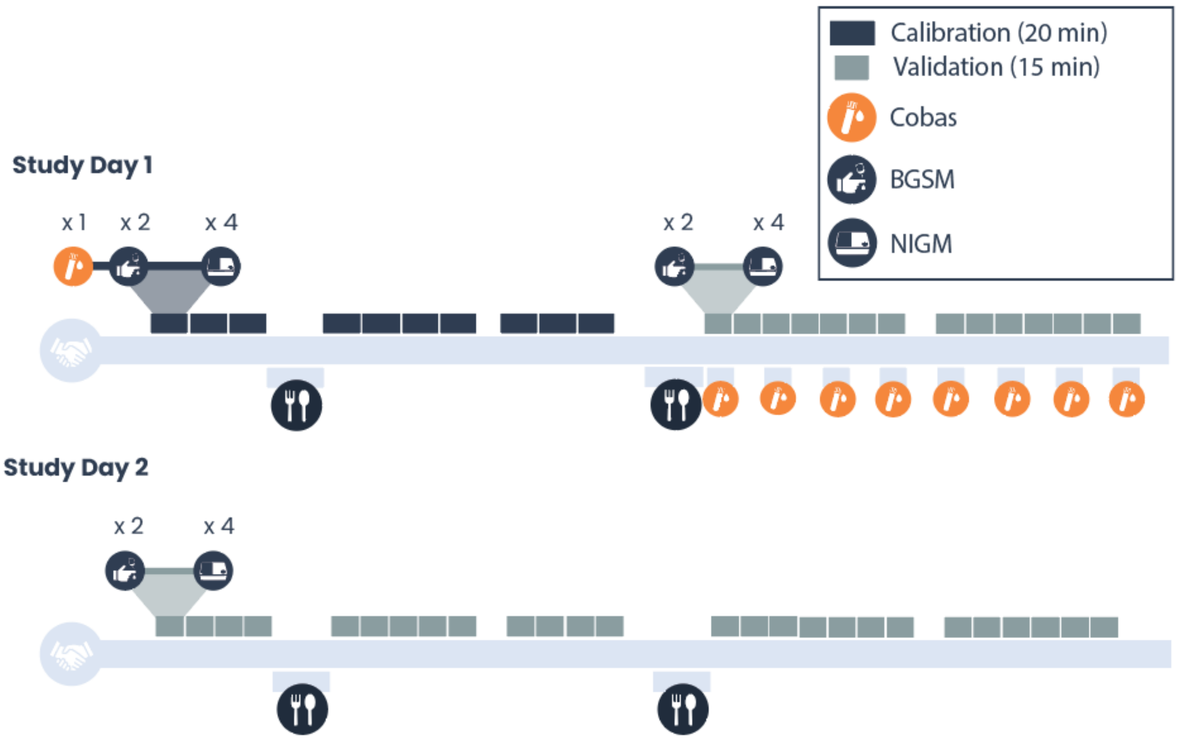

Supplementary Figure 1. Schematic presentation of the 2-day clinical study, highlighting the calibration and validation phases, meals, and breaks. Each block corresponds to a measurement session, involving measurements on NIGM device and BGSM reference glucose value.

Supplementary Figure 2

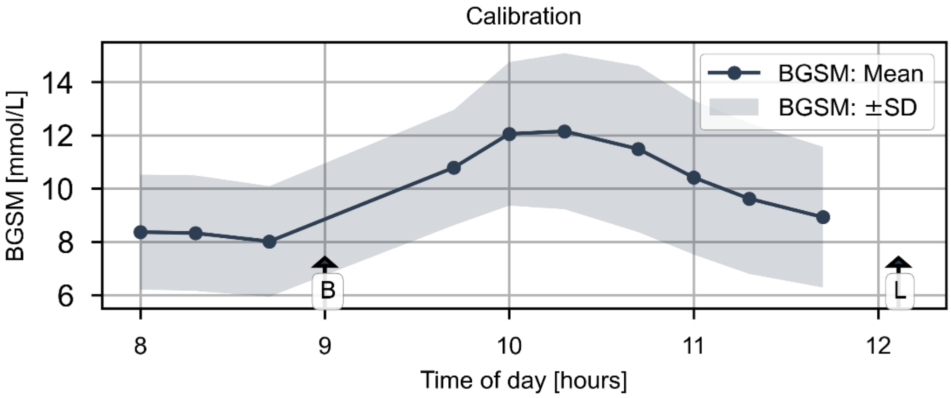

Supplementary Figure 2. Time-course of glucose values during the 4-hour calibration phase in the morning of day 1. The glucose variation is achieved by serving a carbohydrate-rich breakfast (B). L refers to lunch.

Supplementary Figure 3

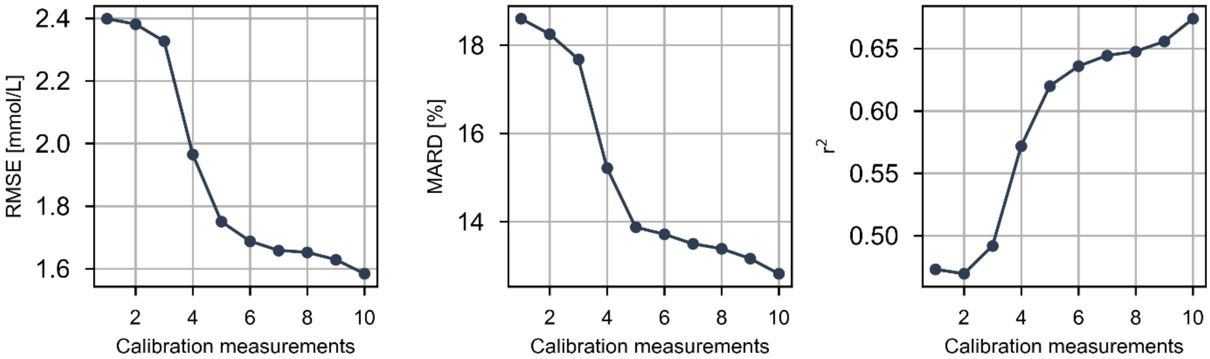

Supplementary Figure 3. Pooled measurement performance vs number of calibration measurements. The measurement performance is described by RMSE, MARD, and squared correlation coefficient r^2. The reduction in number of calibration measurements is achieved by removing measurements that are timewise farthest away from the start of the validation period.

Supplementary Figure 4

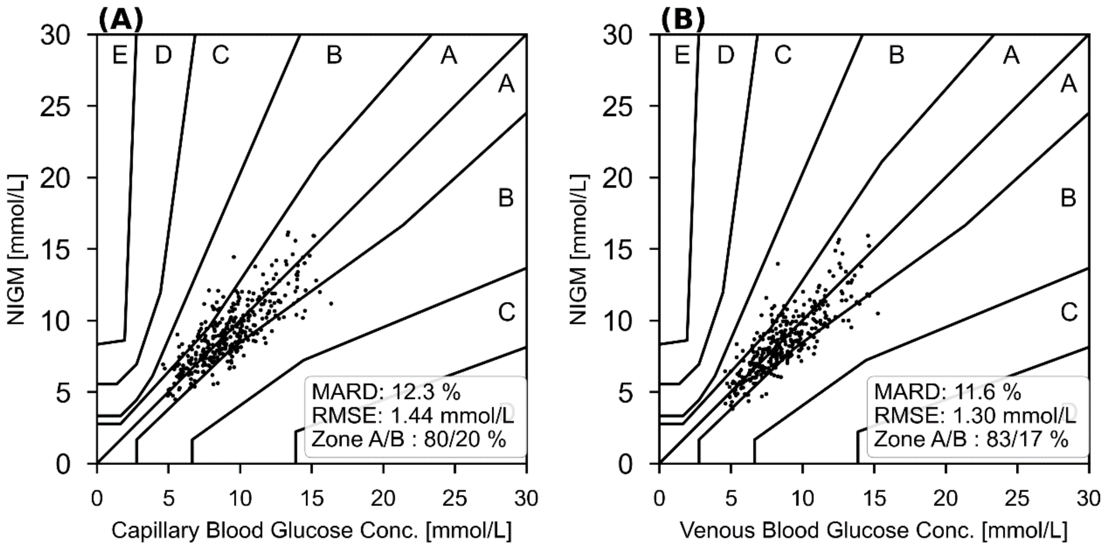

Supplementary Figure 4. Pooled glucose measurements for two reference methods. The NIGM device is calibrated and validated using (A) the home-use Contour Next device and (B) laboratory-grade Cobas system. The simultaneous glucose reference measurements are conducted on a subset of measurements on day 1 (see Supplementary Figure 1).

Supplementary Figure 5

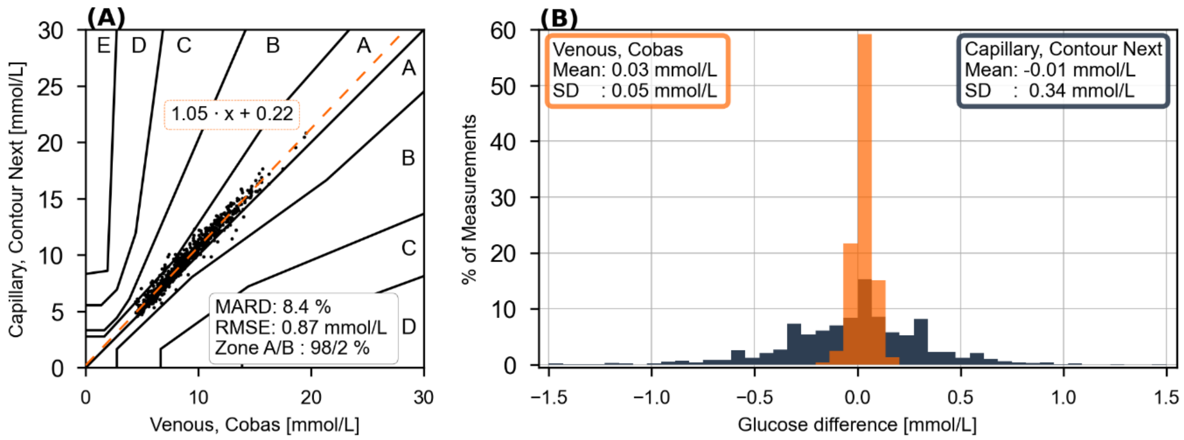

Supplementary Figure 5. Reference glucose values in venous and capillary blood. (A) Consensus Error Grid of capillary blood glucose values (measured with the Contour Next device) vs venous counterparts (measured with the Cobas system). The values are an average of two repeated measurements, and the dashed (orange) line is a linear fit to the paired points, highlighting the systematic difference in glucose values in the two blood compartments. (B) Histogram of the difference in measured glucose values between the two repeated measurements for the two reference methods. The much narrower distribution of the Cobas system underlines its superior measurement accuracy.

Supplementary Table 1

Supplementary Table 1. Correlation between participant characteristics and MARD values. The correlation is considered significant when p < 0.05.

**Variable**		**Pearson correlation, r**	**p-value**
Age (years)		-0.04	0.75
Sex		-0.11	0.44
BMI (kg/m2)		-0.16	0.26
Duration of diabetes (years)		0.19	0.17
HbA1c (mmol/mol)		-0.06	0.69
Treatment	Basic (i.e.: diet, exercise, training)	-0.13	0.35
	Oral antidiabetics	0.26	0.07
	Subcutaneous antidiabetics	-0.01	0.96
	Basal insulin	-0.02	0.90
	Conventional insulin therapy	0.12	0.40
Skin phototype I/II/III/VI/V/VI		0.26	0.07
Stratum corneum thickness (µm)		0.05	0.70
